# Aligning Medication Reconciliation and Secure Messaging: Qualitative Study of Primary Care Providers’ Perspectives

**DOI:** 10.2196/jmir.2793

**Published:** 2013-12-02

**Authors:** Leonie Heyworth, Justice Clark, Thomas B Marcello, Allison M Paquin, Max Stewart, Cliona Archambeault, Steven R Simon

**Affiliations:** ^1^Veterans Affairs Boston Healthcare SystemSection of General Internal MedicineBoston, MAUnited States; ^2^Veterans Affairs Boston Healthcare SystemCenter for Healthcare Organization and Implementation ResearchBoston, MAUnited States; ^3^Brigham and Women's HospitalDivision of General Medicine and Primary CareBoston, MAUnited States; ^4^Veterans Affairs Boston Healthcare SystemPharmacy DepartmentBoston, MAUnited States; ^5^Veterans Affairs Boston Healthcare SystemNew England Veterans Engineering Resource CenterBoston, MAUnited States

**Keywords:** medication reconciliation, secure messaging, secure email, primary care, provider experiences, health information technology (HIT)

## Abstract

**Background:**

Virtual (non-face-to-face) medication reconciliation strategies may reduce adverse drug events (ADEs) among vulnerable ambulatory patients. Understanding provider perspectives on the use of technology for medication reconciliation can inform the design of patient-centered solutions to improve ambulatory medication safety.

**Objective:**

The aim of the study was to describe primary care providers’ experiences of ambulatory medication reconciliation and secure messaging (secure email between patients and providers), and to elicit perceptions of a virtual medication reconciliation system using secure messaging (SM).

**Methods:**

This was a qualitative study using semi-structured interviews. From January 2012 to May 2012, we conducted structured observations of primary care clinical activities and interviewed 15 primary care providers within a Veterans Affairs Healthcare System in Boston, Massachusetts (USA). We carried out content analysis informed by the grounded theory.

**Results:**

Of the 15 participating providers, 12 were female and 11 saw 10 or fewer patients in a typical workday. Experiences and perceptions elicited from providers during in-depth interviews were organized into 12 overarching themes: 4 themes for experiences with medication reconciliation, 3 themes for perceptions on how to improve ambulatory medication reconciliation, and 5 themes for experiences with SM. Providers generally recognized medication reconciliation as a valuable component of primary care delivery and all agreed that medication reconciliation following hospital discharge is a key priority. Most providers favored delegating the responsibility for medication reconciliation to another member of the staff, such as a nurse or a pharmacist. The 4 themes related to ambulatory medication reconciliation were (1) the approach to complex patients, (2) the effectiveness of medication reconciliation in preventing ADEs, (3) challenges to completing medication reconciliation, and (4) medication reconciliation during transitions of care. Specifically, providers emphasized the importance of medication reconciliation at the post-hospital visit. Providers indicated that assistance from a caregiver (eg, a family member) for medication reconciliation was helpful for complex or elderly patients and that patients’ social or cognitive factors often made medication reconciliation challenging. Regarding providers’ use of SM, about half reported using SM frequently, but all felt that it improved their clinical workflow and nearly all providers were enthusiastic about a virtual medication reconciliation system, such as one using SM. All providers thought that such a system could reduce ADEs.

**Conclusions:**

Although providers recognize the importance and value of ambulatory medication reconciliation, various factors make it difficult to execute this task effectively, particularly among complex or elderly patients and patients with complicated social circumstances. Many providers favor enlisting the support of pharmacists or nurses to perform medication reconciliation in the outpatient setting. In general, providers are enthusiastic about the prospect of using secure messaging for medication reconciliation, particularly during transitions of care, and believe a system of virtual medication reconciliation could reduce ADEs.

## Introduction

Adverse drug events (ADE) are common, costly, and preventable [1-3]. Medication discrepancies, defined as unintentional differences between medication listed in the patient’s medical record compared with medication the patient reports taking, are a type of medication error and an important contributor to adverse outcomes [4]. Serious, preventable medication discrepancies are associated with 7000 deaths annually [2]. In 2006, The Joint Commission, an independent organization responsible for accrediting many health care organizations and programs in the United States, designated medication reconciliation as a National Patient Safety Goal [5]. Concurrent with this emphasis on medication safety, there has been a marked increase in the use of patient Web portals and secure messaging (also known as secure email) for patient communication [6]. Innovative organizations are beginning to leverage these tools to improve medication safety and health outcomes [7-10].

Efforts to reduce medication discrepancies using health information technology (HIT) have recently emerged [11,12]. Prior studies using HIT to target medication reconciliation have largely focused on computerized tools for provider-facilitated medication reconciliation during hospitalization [9,13] or in-person medication reconciliation at outpatient clinics [14]. Incorporating HIT in medication reconciliation can reduce potential ADEs and medication discrepancies at the point of hospital discharge [9,15]. ADEs occur at least as frequently in ambulatory care [16], where medically complex patients or those with limited mobility may be at high risk for ADEs because of difficulty accessing primary care services [17].

Strategies for virtual (non-face-to-face) medication reconciliation have the potential to reach patients during vulnerable periods, such as following hospital discharge, when the risk of ADEs is high [5]. Though research has been limited, some studies have found that patients and providers are enthusiastic about exchanging health information via electronic communication [18-21]. In contrast, other studies have suggested less enthusiasm, particularly among providers [22]. The concept of integrating medication reconciliation with secure messaging (ie, secure email between patient and clinical team within a patient Web portal) is appealing, though little literature exists on the topic. One study found that ambulatory patients were satisfied following the use of a Web portal tool for medication management [23]. However, provider perspectives on technology designed to improve medication reconciliation in ambulatory care have received little attention.

In the United States, the Institute of Medicine and the Agency for Healthcare Research and Quality, among others, advocate for restructuring processes of care to emphasize patient-centeredness to improve quality [24,25]. Eliciting primary care providers’ experiences of medication reconciliation can help to inform organizations designing patient-centered solutions for improving ambulatory medication safety. Therefore, in preparation for a pilot study of secure messaging (SM) for medication reconciliation [26], we conducted structured observations of current practices of secure messaging and medication reconciliation in the primary care setting and in-depth interviews among primary care providers. Our aim was to characterize providers’ experiences with medication reconciliation and SM and to characterize providers’ perspectives on a virtual medication reconciliation system, such as one using SM, on ambulatory medication safety.

## Methods

### Setting, Study Design, and the Study Team

This study was conducted at a single Veterans Affairs (VA) medical center with seven associated outpatient clinic facilities. Approximately 39 providers practice in these facilities. To understand the current status and best practices of outpatient medication reconciliation and the potential role for secure messaging in medication reconciliation, the study was designed in two parts: direct observations of medication reconciliation and secure messaging workflow in the primary care clinics, followed by in-depth interviews with providers.

Providers were recruited for this study from January 18, 2012 to May 30, 2012. To be eligible, providers practiced ambulatory primary care and had at least one year of clinical experience at the VA. A nurse informaticist (TM) assisted the study team in identifying the two clinic sites most active in the use of secure messaging. A list of eligible providers at these sites was generated and randomly scrambled in their order; several additional providers were randomly added to this list so that providers from the largest clinic site (and third most active in secure messaging) were represented. Providers were then contacted sequentially via email regarding their willingness to participate in an interview. In this process, only one provider declined. Interviews were conducted until theoretical saturation was reached; a total of 15 providers participated.

The inter-professional study team included two physicians (LH and SRS), two research assistants (JC and TM), a pharmacist (AP), a project coordinator (MS), and a systems engineer (CA).The VA Boston Institutional Review Board approved this study.

### Direct Observations of Secure Messaging Use

To confirm that providers were already engaging in medication management via SM and to better understand the team-based approach to SM triage in primary care, we conducted observations of how primary care staff and providers managed SM at the two largest clinics within the VA Boston Healthcare System. Four individuals on the study team (TM, AP, MS, and CA) led the observations of staff members (providers, nurses, and pharmacists) within two primary care clinics. To minimize observers’ bias, LH and CA created an observation protocol to guide each observer to report on the role and purpose of the staff member replying to the SM, as well as detailing SM workflow and actions taken to process each SM (see Multimedia Appendix 1). On the day prior to the observation, we contacted clinic staff members via telephone or email to explain the intended process of observation. We obtained in-person verbal consent from staff members on the day of observation. Each clinic site was observed once for 2-3 hours by two members of the study team, during which time each observer took field notes while staff members viewed and handled the SM in their inbox at the time. Specifically, we observed how staff members managed secure messages in their inbox, noting how the staff members routed messages to other members of the clinical team, how they referenced other information to respond to patients’ inquiries (eg, medication list or schedule of upcoming visits, both of which are found electronically in a location separate from the secure messaging portal), and how they responded by reply message or by telephone to the patients’ requests.

### In-Depth Interviews With Primary Care Providers

To characterize medication reconciliation practices, we conducted in-depth interviews with 15 eligible primary care providers. An interviewer (LH) obtained informed consent and conducted the interviews using a semi-structured interview guide that we developed (see Multimedia Appendix 2). The interview questions consisted of both closed-ended and open-ended questions (eg, “Are adverse drug events a significant cause of morbidity/mortality for your patients?” and “What does medication reconciliation mean to you?”). Probing questions were added to the script and improvised during the interview to enrich providers’ responses. Each interview lasted approximately 40 minutes. Interviews were structured around three main domains of interest: (1) clinician perspectives on medication reconciliation and adverse drug events, (2) practice of medication reconciliation in the ambulatory setting, and (3) use of, and potential for, secure messaging in medication reconciliation.

Providers were encouraged to make additional open-ended comments about their experiences. A total of 14 interviews were audio recorded; for one interview, the clinician’s responses were transcribed verbatim by hand per request. All recorded interviews were transcribed (JC, TM, and MS) for subsequent coding and analysis.

### Data Coding and Analysis

A co-investigator (CA) compiled handwritten notes from roughly 3 hours of direct observations based on the protocol (Multimedia Appendix 3). We incorporated findings from the structured observations of primary care practices in our analyses of the interview content.

Over ten hours of formal interviews produced 254 pages (double-spaced) of transcription. All transcripts were de-identified prior to coding. We conducted a content analysis informed by grounded theory, with the goal of developing a theoretical framework for using secure messaging for medication reconciliation [27,28]. The initial coding scheme was developed by a team (LH, JC, and SRS) from a sample of three of the interview transcripts: LH and JC independently reviewed these transcripts, annotating important themes, and then met with SRS to review the themes and generate a coding scheme. The newly defined coding scheme was then applied to the remaining transcripts (JC and TM) with regular team meetings to discuss discrepancies and to refine the coding scheme. Discrepancies were resolved by consensus. All coded transcripts were reviewed to ensure that the quotes selected were relevant and accurate to the established coding scheme (LH and TM). We used NVivo 8 for coding and analysis of the interview data [29].

## Results

### Observations of Secure Messaging in Primary Care

**Figure 1:**
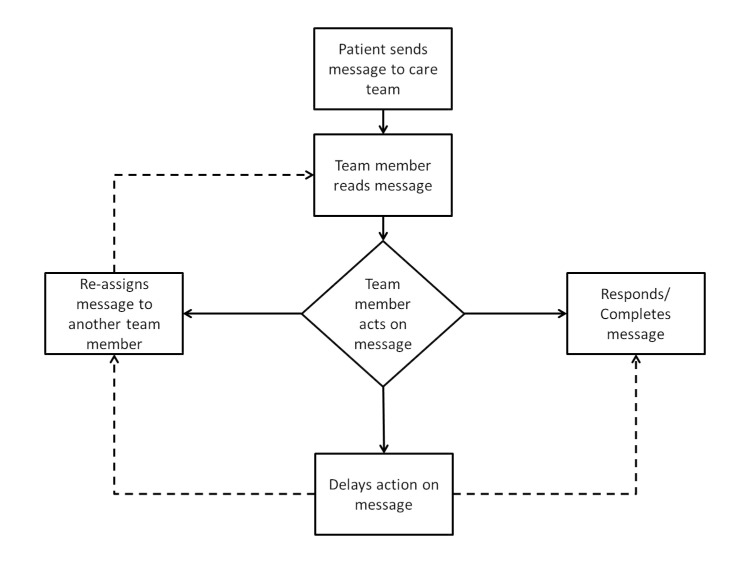
Observations of secure messaging workflow in primary care. This diagram depicts the observed sequence of events by the clinical team in response to retrieving an SM (42 SM were observed in total).

Our objectives were to ascertain which members of the clinical team were viewing secure messages from patients, how messages were forwarded among staff members, and the workflow for resolving the requests or issues raised in the patient’s message. We also wanted to confirm that SM was being used to address issues related to medication management—a topic we hoped providers could address in detail during the in-depth interview. In observations of SM use within primary care, we observed that the first member of the clinical team to view each of the 42 observed patient-initiated SM were divided between 5 primary care providers (physicians or nurse practitioners), 4 licensed practicing nurses, 3 registered nurses, and 2 pharmacists. Among 42 SM managed by staff members during our structured observations, the most common subjects of patients’ secure messages related to medication management (20 messages) and general medical issues (7 messages). Among the 42 messages observed, the individual initially retrieving the message most commonly responded or completed the message immediately (27 messages) and less commonly reassigned the message to a more appropriate person (9 messages). In a minority of cases, there was a delay of more than two business days before responding to the patient’s message (6 messages). The observed workflow, illustrated in Figure 1, provided a foundation for creating relevant in-depth interview questions. Appreciating the team-based structure of SM triage and confirming that medication management was already being conducted were common experiences upon which providers could further elaborate during the interview.

### In-Depth Interviews With Primary Care Providers: Baseline Characteristics

We interviewed 15 eligible primary care providers about their medication reconciliation practices and perspectives on the use of secure messaging. Twelve of the providers interviewed were female and 10 worked at the largest primary care clinic within the local VA system. Eleven providers saw 10 or fewer patients in a typical workday. All providers reported having staff to assist with patient-care responsibilities and all providers deferred to a hospitalist for inpatient care of their patients. Providers consistently reported that medication reconciliation, based on their own definition, was performed less than 25% of the time prior to a patient encounter. Only one provider noted that her practice tracked adverse events. All but one provider recognized that the Computerized Patient Record System (CPRS), the VA’s electronic health record, included computerized decision support to improve medication safety. Comparing providers interviewed for our study with the demographics of other providers working at these same three clinic sites, providers in our study were more likely to be a physician, female, and practice at the two clinic sites closest to VA Boston’s hospital; study participants reflected the larger population of providers in this setting in terms of deferring to an inpatient physician for care of inpatients (Table 1).

**Table 1:** Characteristics of providers participating in an interview, compared to providers within the VA Boston Healthcare System.

Characteristics		Study Participants, n (%)	All VA Boston Primary Care, n (%)	*P* value^a^
**Providers**				
	MD	13 (87)	31 (79)	.45
	Nurse Practitioner or Physician Assistant	2 (13)	8 (21)	
**Gender**				
	Female	12 (80)	25 (64)	.17
	Male	3 (20)	14 (36)	
**Clinic Site** ^b^				
	Jamaica Plain	9 (60)	15 (38.5)	.002
	West Roxbury	6 (40)	9 (23)	
	Brockton	1 (7)	15 (38.5)	
**Has staff to assist with patient-care**				
	Yes	15 (100)	-^c^	-
	No	0	-	
**Attending physician of record when patients are admitted** ^d^				
	Yes	0	0	-
	No	14 (93)	31 (100)	
**% of patients who had medication reconciliation performed prior to office visit**				
	0-24%^e^	15 (100)	-	-
	25+%	0	-	
**Uses decision making or support tools to prevent adverse drug events**				
	Yes	14 (93)	-	-
	No	1 (7)	-	

^a^ *P* values calculated via Fisher’s Exact Test.

^b^Does not sum to 15 due to 2 physicians with clinics at 2 sites.

^c^“-“ denotes an absence of a system-wide standard practice.

^d^Numbers may not sum to 15 due to non-response.

^e^Physicians who reported “few”, “rare”, “not usually”, “very few”.

### Provider-Reported Experiences and Perspectives in Outpatient Medication Reconciliation

Four overarching themes characterized providers’ experiences and perspectives with medication reconciliation in the primary care clinic (summarized in Table 2). First, providers shared the belief that good quality medication reconciliation could improve outcomes. Second, providers felt that achieving quality medication reconciliation among medically complex patients was often challenging. Third, providers identified systems-level obstacles that prevented high-quality medication reconciliation. Last, providers described opportunities they believed could improve medication reconciliation after hospital discharge. Each theme is examined further, using providers’ quotes to illustrate subthemes.

**Table 2:** Summary of themes on provider-reported experiences and perspectives in outpatient medication reconciliation.

**Theme 1: When done right, medication reconciliation can be effective**	
	Medication reconciliation has the potential to improve medication safety
	No standard approach to quality medication reconciliation
**Theme 2: Perceived patient-level challenges to effective medication reconciliation**	
	Patients often lack understanding of their medications
	Home environment of patients often chaotic
	Informed caregivers are valuable in assisting with medication reconciliation in complex patients
**Theme 3: Perceived system-level obstacles preventing high-quality medication reconciliation**	
	Limited time and staff support for medication reconciliation
	Lack of subspecialist involvement in managing medications they prescribe
	EHR^a^ often complicates medication reconciliation
**Theme 4: Perceived opportunity for improving medication reconciliation after hospital discharge**	
	Medication reconciliation is already a key priority during the post-hospital follow-up visit
	Leveraging EHR capabilities for medication management
	Team-based collaborative care for improving medication reconciliation

^a^EHR: electronic health record

### When Done Right, Medication Reconciliation Can Be Effective

#### Overview

Most providers believed that performing in-depth, quality medication reconciliation could have the potential to decrease ADEs and improve medication safety in general, though many providers acknowledged the lack of a standard approach to medication reconciliation.

#### Medication Reconciliation has the Potential to Improve Medication Safety

Providers emphasized the importance of performing medication reconciliation in order to recognize side effects of medications and their interactions, even when medications are taken as prescribed:

I think [the effectiveness of medication reconciliation] is huge…because the side effects of various meds in poly-pharmacy are frequently…more deleterious for the patients than the illnesses the medications were prescribed for.

Provider 3

I think what people do for medication reconciliation is so variable…if you really do it as a process, it would be highly effective. Some adverse drug events [are] just the risk of [taking] medicines, [it] is never going to be zero, but when done right, [medication reconciliation can be] highly effective.

Provider 5

#### No Standard Approach to Quality Medication Reconciliation

Limited formal training in conducting medication reconciliation was commonly cited and many providers had not adopted a standard approach for in-person medication reconciliation:

Honestly, I, once in a while, might read an article about it, in a journal, but most of my education just comes through the discussion we have in our primary care meetings.

Provider 9

### Perceived Patient-Level Challenges to Effective Medication Reconciliation

#### Overview

Providers shared concerns regarding experiences where patients lacked skills in medication management. This issue was magnified by disorganized home environments and particularly challenging for medically complex patients, where providers reinforced the value of a caregiver to corroborate the medication list.

#### Patients Often Lack Understanding of Their Medications

Providers suspected that a significant proportion of their patient population both lacked understanding of their medications and did not take their medications as prescribed:

It’s probably 50/50 [of patients accurately taking their medications]. I do see a lot of young women who are [taking] just a few medicines and are very knowledgeable...And then I have a handful where, sometimes it takes multiple visits and even this morning, calling a visiting nurse and really trying to figure out who’s putting the pills in the box, and what exactly is going in there.

Provider 5

I mean, I think I’m a very good clinician, but I bet it’s probably only 30 or 40% [of patients accurately taking their medications]. It’s probably not very good.

Provider 15

I think that what is useful sometimes is when they actually bring their bottles in with them. But then again sometimes the bottles can be expired, they can be from other physicians who prescribed them, and if they aren’t sure what they are taking even with the bottles, then it can be even more challenging.

Provider 9

#### Home Environment of Patients Often Chaotic

Many providers suspected or were aware of poor medication organization at home and cited this as being a major barrier to accurate in-person medication reconciliation:

And an example recently is someone finally went into the home of [a patient] only because their diabetes wasn’t under good control and the person agreed to a home visit. And this guy is functioning and working, they found pill bottles old, new, mixed up in every room of the home. No one had any idea that it was going to be that chaotic.

Provider 12

I have patients who I don’t know the full list of what they have at home, but they will tell me I have some [medications]…but won’t throw them away. Then it’s really impossible to do medication reconciliation because I can’t even begin to understand what they have at home.

Provider 5

#### Informed Caregivers are Valuable in Assisting With Medication Reconciliation in Complex Patients

Providers frequently described that medication reconciliation among medically complex patients, such as those with multiple chronic conditions or dementia was challenging. Presence of a caregiver or someone who had knowledge of the patient’s medication administration in this situation was felt to be very helpful in achieving accurate medication reconciliation.

It helps if there’s a caregiver or someone in the family who comes with them…sometimes you can’t complete medication reconciliation with the veteran themselves in that situation, so you have to rely on caregivers.

Provider 1

So I usually try to always have them bring the pill bottles. Then I always try to get collateral information. Usually they’re in an assisted living or in a setting where other people are also involved. I try always to contact who else is involved in the administration of the medications, to really understand how they are taking it.

Provider 5

### Perceived System-Level Obstacles Preventing High-Quality Medication Reconciliation

#### Overview

For many providers, issues relating to organizational structure or clinic workflow frequently impeded their ability to perform effective medication reconciliation. Providers repeatedly expressed frustration in managing medications prescribed by specialists or non-VA providers and many felt that the EHR medication reconciliation more difficult to accomplish than expected.

#### Limited Time and Staff Support for Medication Reconciliation

The majority of providers felt that the time provided in a routine primary care appointment was often insufficient for detailed medication reconciliation. Providers recognized that executing medication reconciliation could be especially time-intensive among cognitively impaired patients. Many providers acknowledged a lack of support staff to assist with medication reconciliation, primarily taking on this task alone:

A pharmacist doing [medication reconciliation] in-person with the patient and the family when they are around at the time of discharge takes an hour per patient to do med rec. So how they expect, in a 30-minute follow up visit with a complicated patient, that this is going to get done…and the estimates from the nursing staff is 40% of the discharged patients are cognitively impaired.

Provider 8

It depends on the number of medications, of course. And it depends on the detail in which we are doing medication reconciliation. A very cognitively impaired person, who’s living independently, is going to take a lot more time, because we are going to look in the bottles and potentially do some pill counts to check.

Provider 13

My nurse or LPN hands me a list with the veteran’s printed medications on them and I would say one out of ten times there are checkmarks on this document indicating some kind of [medication] discrepancy, but would I call this medication reconciliation? No.

Provider 14

#### Lack of Non-VA or Subspecialist Provider Involvement in Managing Medications They Prescribe

A commonly cited experience among providers was the responsibility for managing medications prescribed by subspecialist physicians adding to the challenge of accurate medication reconciliation in primary care:

I think a lot ends up falling on primary care…we’re often called upon to reconcile things that we’re not necessarily managing. So it has to be something that the whole medical center buys into so that we can get the help of subspecialists…we might not be able to resolve the discrepancies ourselves.

Provider 1

I think that what is useful sometimes is when they actually bring their bottles in with them. But then again sometimes the bottles can be expired, they can be from other physicians who prescribed them, and if they aren’t sure what they are taking even with the bottles, then it can be even more challenging.

Provider 9

#### EHR Often Complicates Medication Reconciliation

Providers reported difficulty clearly displaying reconciled medications in the EHR. This issue was often magnified among patients being seen in subspecialist clinics or care outside of the VA:

It bothers me that specialty clinics don’t change the med list in [the EHR]. If they do add a new medicine to [the EHR], it automatically spits out a new refill, which may not be what the veteran needs at that moment. So, the specialist often does nothing, which makes it difficult for me to figure out what the actual dose or medicine they are taking is. I wish specialists could just update the medications in CPRS without it automatically generating a refill.

Provider 14

And then we have people who literally are here to save $5 a month on their simvastatin and it’s a completely different situation… they are really coming in for medication reconciliation, updating our medical records, making sure they have recent labs so we can safely give them the cheaper medicines. It’s almost like a pharmacy transaction and less of a medical visit, which is why so many VA [physicians] hate [medication reconciliation]. It just doesn’t feel that good.

Provider 2

### Perceived Opportunity for Improving Medication Reconciliation After Hospital Discharge

#### Overview

A number of providers described potential solutions for improving medication safety during a vulnerable transition period, such as following hospital discharge. Providers commonly suggested technology-based solutions involving the EHR and also believed that training support staff to assist with medication reconciliation could improve the process.

#### Medication Reconciliation is a Key Priority During the Post-Hospital Follow-Up Visit

There was agreement among all providers that medication reconciliation was a priority when seeing a patient in follow-up of a hospital visit:

Medication reconciliation is usually the first priority. Oftentimes, things have been changed in the hospital reflecting things that were going on there, when it’s better just to have people on their pre-hospital medication regimen. I think that medication reconciliation is…probably the first priority.

Provider 11

#### Leveraging EHR Capabilities for Medication Management

Providers imagined a variety of approaches to improve medication reconciliation, many involving streamlining the EHR to identify errors and interactions:

The other part of that is that I think there can be electronic surveillance of medications or a mechanism to identify those patients who aren’t refilling. That’s probably a pretty good clue if they haven’t refilled in 6 months…I think that is something that is within our grasp as well.

Provider 13

I personally think the more you can do on the online automated system the better. For example, if you prescribe someone a potentially dangerous medication [and]…there is no follow up within a certain amount of time, you should not be able to discharge patient.

Provider 4

#### Team-Based Collaborative Care for Improving Medication Reconciliation

A majority of providers envisioned a scenario where a pharmacist or clinical staff member performed detailed medication reconciliation prior to the provider’s visit. This could minimize the time necessary for medication reconciliation by the provider, freeing up time to discuss clinical issues:

In an ideal world, it would be awesome if, when the patient comes in, or that time when they are sitting in the waiting room, there was a pharmacist or someone who could do that medication reconciliation process in a way so that when they do make it into the exam room, I know the list already…that would be in an ideal, perfect world, to already have that done, before I even see them.

Provider 5

I don’t think it is the physician’s responsibility to do the whole extensive med rec, I think it’s very time consuming and you will never get to the other clinical issues and that is really what you are trained to do. So I envision nurses and clinical pharmacists really can do this better.

Provider 12

I would love [for pharmacists] to review the prescriptions with the patient after the visit. If there was…a real problem patient… If I could say to them, look I’m really having a problem with this patient. He brought all his medications in but he’s also in congestive heart failure or he’s worse or whatever problem I might need to deal with that day, could you go over his meds with him?

Provider 15

I think for certain patients, it would be helpful to have a system where they did bring in their pills and maybe reviewed them with a nurse ahead of time and then the nurse kind of made note of where the discrepancies are and brought that to me and then I could further work on where the discrepancies are.

Provider 1

### Providers’ Experiences Regarding Secure Messaging

We identified 3 main themes with respect to providers’ SM use (summarized in Table 3): (1) the use of SM improved workflow, (2) the existence of patient and provider-level barriers to use of SM, and (3) providers believed in the potential of using SM to improve medication reconciliation in ambulatory primary care. Each theme is described in detail, with selected quotes exemplifying each theme.

**Table 3:** Summary of themes on providers’ experiences regarding secure messaging.

**Theme 1: Improved work flow**	
	Avoids “phone tag”
	Increases workflow efficiency
**Theme 2: Obstacles to more frequent use of secure messaging**	
	Technical difficulties accessing secure messaging
	The process of enrollment and use of SM can be complicated for patients
**Theme 3: Integrating medication reconciliation and secure messaging**	
	Secure messages as potential tool to assist with reconciling medicines in the outpatient setting
	Potential to decrease adverse drug events

### Improved Work Flow

#### Overview

Providers who used SM agreed that many patient questions and requests were streamlined and addressed by the most appropriate member of the team.

#### Avoiding “Phone Tag”

The direct patient contact via SM reduced time spent in “phone tag” (ie, leaving messages for the patient to call back) and providers reported feeling like communication was easier and often more descriptive.

The electronic communication is wonderful. It avoids the whole issue of phone tag. It avoids the whole issue of someone having to give their message to another person, which often distorts the meaning of the request. The asynchronous communication makes all communication easier.

Provider 2

You can get patients’ answers to questions in a more timely fashion than playing phone tag, so it can be a little bit more. You can have a back-and-forth conversation and get work done and document the work that you’re doing.

Provider 1

#### Increases Workflow Efficiency

The team-based model of SM triage means that providers never saw many of the messages that patients addressed to them, as team members were able to answer and fulfill requests by SM with minimal or no provider input, something providers appreciated.

Patients typically will communicate with me first to let me know that they need something. My team screens all the incoming secure messages and they only give it to me if it’s a medication renewal. They screen all the messages so I don’t have to deal with certain things that don’t need my involvement.

Provider 15

I think it can also get to the point of it much more succinctly. Not to be antisocial, but you don’t have to deal with the niceties of ‘How are you feeling today?’ They write you with whatever is the concern and you respond to it.

Provider 10

It’s helpful because it’s taken away the clutter from the call center in my inbox and all the things I don’t need to deal with like appointments and physical therapy consults are taken care of.

Provider 14

### Obstacles to More Frequent Use of Secure Messaging

#### Overview

Providers expressed frustration with their inability to easily access SM and also identified the multi-step process for SM registration and less technically savvy elderly patients as potential challenges to widespread patient adoption of SM.

#### Technical Difficulties Accessing Secure Messaging

A commonly cited complaint among providers was the need for a separate log-in to the SM service and slow network speeds:

It’s a very difficult system to use. It’s often…not working and it’s not easy to get into…If I need to use secure messaging…I have to look [the patient] up and wait and wait for the delay…I know they are planning to interface it better with our Outlook in the future.

Provider 7

I think the speed of our network is horrible and so that, yet another password, yet another log-in. My computer takes 20 minutes to boot in the morning, we’ve timed it….I have to check my emails and check my secure messages, yet another administrative burden. And my value should be in talking to patients, seeing patients, or personally writing papers and developing…

Provider 13

#### The Process of Enrollment and Use of SM Can Be Complicated for Patients

Providers commonly noted that a lengthy opt-in process, the requirement for in-person authentication, as well as limited technical literacy of many elderly patients as barriers to increased SM use among patients:

You have to remember our patient population isn’t a high-tech patient population. I think [using SM for the elderly] would be relatively ineffective.

Provider 13

it looks like we have a lot of patients who got authenticated, but haven’t opted in…I think the opt-in process is not clear to patients. It shouldn’t be so hard to check a box accepting terms and conditions. A lot of the people who have been opted in are younger, tech-savvy veterans. If this were user friendly, they would be able to do it. They shouldn’t need a lot of coaching.

Provider 2

### Integrating Medication Reconciliation and Secure Messaging

#### Overview

Providers were uniformly enthusiastic about the potential for SM to improve medication reconciliation in primary care, particularly in the post-hospital discharge period and in clinics without the help of a clinical pharmacist.

#### Secure Messaging: Potential Tool to Assist With Reconciling Medicines in the Outpatient Setting

Having acknowledged the vulnerability to complications during transition periods such as post-hospital discharge (see above), providers reported that SM could also play a role during such a time:

I think [SM] would be helpful…there are a lot of problems with med rec through the transition.

Provider 12

Providers also expressed the potential of SM to fill the void in clinics not assigned a clinical pharmacist to assist with medication management:

[Using SM for medication reconciliation] would solve the problem of not having an available pharmacist. It’s just a huge disparity in access to services that other clinics should have the full-time pharmacist and we should have nothing.

Provider 2

#### Potential to Decrease Adverse Drug Events

All providers believed that SM had the potential to reduce ADEs. Proactive and frequent medication reconciliation was among providers’ most common reasons to believe that medication reconciliation by SM could reduce ADEs:

Oh, I think [using secure messaging for medication reconciliation] would make [ADE] go way down, because you would be more proactive about finding the problems, instead of waiting until the adverse event happened and then discovering the problem.

Provider 4

Using [secure messaging for medication reconciliation] engages the patient a little bit more. And I feel like the more the patient is aware of their medicines and the potential for adverse drug events and can identify things sooner on their own, that’s always great. The more knowledgeable they are [of] their medicines and having the interface with the computer would help that. I think it would decrease adverse drug events.

Provider 5

## Discussion

### Principal Findings

In this study across multiple ambulatory clinics within a large VA health care system, we conducted direct observations of medication reconciliation and secure messaging workflow in primary care and interviewed primary care providers about their experiences with medication reconciliation. Our findings confirm providers’ perception that medication reconciliation has the potential to improve medication safety. Providers highlighted a number of patient-level obstacles hindering high-quality medication reconciliation, emphasizing the difficulty in achieving accurate medication reconciliation among complex or elderly patients. Providers identified limited time and support for medication reconciliation as key barriers. While the majority of providers felt that the task of medication reconciliation was the responsibility of the primary care provider, almost all providers favored shifting this often time-intensive task to support staff or pharmacists and suggested that the process could be optimized by being conducted prior to the visit, either virtually or in person.

With respect to secure messaging, providers commonly expressed positive experiences and reported that its use in primary care improved workflow, largely via team-based message triage, but felt that making it easier for patients to sign up for and use SM could foster more frequent provider use and increase patient adoption. We found that providers were optimistic about the potential use of SM for medication reconciliation, primarily seeing such a system as an opportunity to decrease ADEs.

Traditionally, medication reconciliation has been recognized as the responsibility of the prescriber, usually a physician. Our finding that most providers would choose to relinquish this role to pharmacists or share the responsibility with clinical support staff is noteworthy and supports prior literature demonstrating provider support for the role of pharmacists in medication reconciliation [13,30]. This perspective may reflect the increasing pressures in primary care, including a workforce shortage resulting in increasing patient care demands [31], pay-for-performance programs [32], as well as high levels of physician burnout [33]. Future research should focus on providers’ definitions of medication reconciliation and their perception of their role and the role of other team members in executing it.

**Figure 2:**
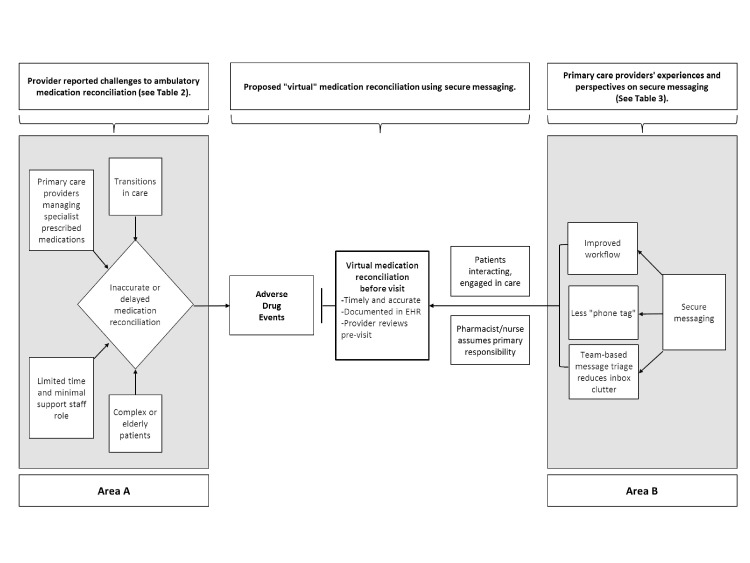
Conceptual model of the challenges to ambulatory medication reconciliation and the possible role of virtual medication reconciliation. Area A shows challenges to medication reconciliation and Area B reflects providers’ perspectives on secure messaging and medication reconciliation. The central area between A and B proposes the possibility of virtual medication reconciliation from home following hospitalization.

Our findings underscore the ongoing challenge of effective medication reconciliation in primary care, particularly for medically complex patients and during transitions in care. Our study suggests that a tool designed to facilitate virtual (non-face-to-face) medication reconciliation via SM would likely benefit providers in primary care (Figure 2).

There is little literature describing effective or novel medication reconciliation practices in primary care [34]. One recent study demonstrated provider interest in a patient self-service kiosk linked to the EHR for medication reconciliation [35], while other studies have shown that existing SM users within the VA system have lower rates of health care utilization [36]. Surveys have consistently found high patient interest and satisfaction ratings when given the option to communicate with providers via SM [37-39]. At a time when large health care organizations are investing in patient portals and Web-based health management [8,40], our study contributes the perspectives of primary care providers to the existing literature that, to date, has focused on patients’ willingness and organizational-level readiness to embrace Web portals and SM to manage care.

### Limitations

There were several limitations of our study. First, interviews were conducted among a small number of individuals (mainly female physicians) at three clinics within a single institution, possibly limiting generalizability. Future research should examine perspectives from other members of the clinical team. One of the interviews was transcribed by the interviewer instead of being recorded (given the wishes of the interviewee), potentially impacting the data collected. However, this interview was conducted over a longer time frame in order to mitigate possible inaccuracies and to transcribe complete quotes. Second, because these interviews were conducted ahead of a pilot study recruiting patients to use a medication reconciliation tool within secure messaging, it is possible that some providers were alerted to this intervention by patients participating in focus groups, priming their perceptions of SM use for medication reconciliation prior to the interview. Future research should examine providers’ perspectives in the proactive use of SM for a range of topics in primary care. Last, all providers were part of a large, integrated national health care system with a common EHR, established patient portal, and computerized provider order entry linked to pharmacy dispensing software, resulting in potentially distinctive provider perspectives on medication reconciliation and SM compared to providers practicing in clinics or private offices without such integration, and thus limiting generalizability.

### Conclusions

With the pursuit of medication reconciliation as a National Patient Safety Goal [5,30,41-43], novel approaches to accurate medication reconciliation will be vitally important to improving medication safety and systems of care within primary care. Our study found that primary care providers, on the frontlines of patient safety and chronic illness management, recognize the importance of complete and accurate medication reconciliation. Providers favor having their professional colleagues, such as nurses and pharmacists, assume primary responsibility for medication reconciliation and express enthusiasm about aligning and shaping SM for the purposes of medication reconciliation. At a time of organizational readiness for patient-led online health management [8,40], future studies should focus on the design and implementation of SM or other Web-based tools for medication reconciliation.

## Multimedia Appendix 1

Secure messaging observations protocol.

## Multimedia Appendix 2

Interview script.

## Multimedia Appendix 3

Coding scheme.

## Abbreviations

ADE: adverse drug event
CPRS: Computerized Patient Record System
EHR: electronic health record
HIT: health information technology
SM: secure messaging
VA: Veterans Affairs
