# Supplementary material for: Aligning Medication Reconciliation and Secure Messaging: Qualitative Study of Primary Care Providers’ Perspectives
Source: J Med Internet Res. 2013 Dec 2;15(12):e264. doi: 10.2196/jmir.2793 (PMC3868963; doi:10.2196/jmir.2793)
Supplement: Supplementary file 1 [file jmir_v15i12e264_app1.pdf]

APPENDIX 1

Secure Messaging Direct Observation

Study Name: Interactive Medication Reconciliation by Secure Messaging

Principal Investigator:

Element of the study: Direct observation of the current use of secure messaging in primary care.

Purpose: To understand the uses for which secure messaging is currently employed in clinical care and the processes of using secure messaging. This is part 2 of a 2 part direct observation study. Part 1 will focus on the current process for medication reconciliation in primary care with emphasis on post discharge medication reconciliation. The two observational studies will inform focus group and interviews, and together the analysis will influence recommendations for a pilot of interactive medication reconciliation through the use of secure messaging.

Protocol

- Prior to day of planned observation:
    - Observer meets with clinic supervisor/leadership to explain the observation process.
    - Observer may join a staff meeting to explain the process and identify staff that use secure messaging.
    - Staff who currently uses secure messaging to interact with patients will be identified and approached to be included in the direct observation of secure messaging.
- Day of visit: Observer explains process to the staff member(s) and clinician(s) to be observed and obtains voluntary consent.
- Who is being observed:
    - Only the employee(s) who gave consent will be observed.
    - Veterans will not be observed during this process; however, observers will be exposed to the patient's secure messages sent to their care team. Patient details of the secure message will not be recorded but the general purpose of the message (appointment request, health condition question, etc will be: only the process is under study.
- Observation notes will be recorded on the worksheet below.
    - Observation worksheets will be stored in a locked cabinet

- No personal health information (PHI) will be collected
- At any time staff member may ask the observer to leave the area and/or stop recording notes. The observer will respond to this request immediately.
- Where observations will take place:
  - Participants will be observed as they perform their usual, work-related activities
    - Participants will not be followed into private areas such as the restroom or dressing room
  - Secure message interactions occur outside of face to face interaction with patients.
- We will not record information that is not relevant to the secure messaging interaction.

Script for introducing ourselves as observers:

1. Introduce yourself & purpose to the staff member

   a. Name & project: "Hi; my name is ___ and I am part of a VA-funded research team studying ways to improve medication list management through the potential use of secure messages. I would like to observe how you use secure messaging today."

   b. What is direct observation: "The purpose of direct observation is to understand the true current reality. Therefore, I would like to request that you act as you would do while managing the secure messages."

2. Seek consent from staff member

   a. Explain what will happen: "I'd like to observe you read and respond to secure messages and ask you questions about when and how you respond. I will ask you to explain each message in terms of who received it first, who else it was sent to, how you determine whether to respond and what to respond, and what you expect next steps will be for this message."

   b. Explain what is confidential: "All information collected will be held confidential. My observation notes will not be made available to your supervisor or anyone else other than our small group of observers. Observation notes will be compiled with those from other clinicians and at other clinics and results will not be linked to your name or other personal identifiers. Any comments you make to me may also be compiled with other feedback from staff but not linked to your name."

c. “Your participation is completely voluntary. There is no penalty if you do not wish to participate, and no personal benefit or compensation if you do wish to participate. You may choose not to answer any clarifying question that is posed, and you can decide to stop participating at any time with no negative effects. Nothing about these observations will be used to judge you personally or to evaluate your performance in your job. “If you have any questions about these observations, please contact Dr. Leonie Heyworth at (857) 364-5704. If you have any questions about your rights as a participant, please contact the Employee Advocate at your facility (857) 364-5562.

d. Get consent: “Is it ok if I observe your secure messaging work today and take notes about the secure message process?”

   i. If yes, proceed

   ii. If no, stop; will need to find a different willing staff member to observe.

Observation Notes

The following is a general outline of the information we intend to collect. Observers will remain alert to any interactions or behaviors in the primary care setting that may be relevant to the medication reconciliation process:

1. Who is doing the secure messaging (staff member role)
2. What is purpose of secure message; who initiated the message (patient or staff)?
3. Prior flow of message (triage, who else received, etc)
4. Time to respond to the message – both the lead time from when the veteran sent the message to reply, and the processing time for the staff member to craft the response.
5. Next steps flow of message (closed, open pending another responder, open pending patient response)
6. Actions taken as part of processing the message (follow up appointment scheduled as a result of secure message, MD/PharmD consulted for content of response etc)
7. Staff concerns about the specific message

8. Problems and/or interruptions

Observer: ________________

Clinic: ___________________ Date: _____________

#	Time per Secure Message	Observed Staff Role & Setting	Purpose of Message & initiator				Observation Notes (interruptions, problems, staff concerns)
1	Days since message sent: __________ Minutes processing response __________						
2	Days since message sent: __________ Minutes processing response __________						
3	Days since message sent: __________ Minutes processing response						

4 Days since message sent:

Minutes processing response
