# Supplementary material for: Aligning Medication Reconciliation and Secure Messaging: Qualitative Study of Primary Care Providers’ Perspectives
Source: J Med Internet Res. 2013 Dec 2;15(12):e264. doi: 10.2196/jmir.2793 (PMC3868963; doi:10.2196/jmir.2793)
Supplement: Supplementary file 2 [file jmir_v15i12e264_app2.pdf]

APPENDIX

Clinician Interview Script

Introduction

The overall goal of this this research is to improve medication reconciliation. Medication reconciliation is often defined as the processes that are used to ensure that patients and those who care for them have a good idea of what medications the patient is supposed to be taking. The purpose of this project is to develop a medication reconciliation system through the My Health*e*Vet secure portal. The purpose of this interview is to better understand current medication reconciliation practices and secure messaging use in order to guide our development of the pilot platform.

We are looking for your honest feelings and opinions; there are no right or wrong answers in this interview and all your responses will be confidential. In any reports of these interviews, we will never mention your name, practice, or other personal information.

Do you have any questions before we begin?

Background

1. I would get a feel for your practice.
    a. At which clinic site do you see patients?
    b. How many providers and staff work at your clinic?

c. What staff directly assist you with patient-care responsibilities? (nurses, physician assistants, health technicians, secretaries, other physicians)

d. For a typical patient, what's the workflow? That is, when a patient comes into your office, tell me about the staff they interact with and the sequence in which these interactions happen. What happens during each of these interactions?

2. Tell me about your patient population.

a. Age group and distribution; gender

b. Approximately how many patients do you see in a day? In a week? Of these patients, how many do you see per week in follow up to a hospital stay?

3. We're interested in learning about your patients who are hospitalized.

a. Do you care for your patients if they are admitted or defer to an inpatient team?

b. Percent of patients admitted infrequently / once per year / multiple times per year

c. Of your patients that are admitted to the hospital, what percent of the time are you aware of this within 48 hours, within 1 week, greater than 1 week? Tell me about the sources by which you find out about patient admission.

d. When your patients are discharged from the hospital, when do you typically hear about their discharge? (As it's being planned? Day of discharge? Sometime after discharge?)

4. We are especially interested in the transition from hospitalization back to your office.
    a. In general, what happens in that transition?
        i. How long of a time period? Is it too long, too short, or just right?
        ii. Who, if anyone, typically communicates with the Veteran in the interim?
        iii. What kinds of issues come to your attention in that transition time?
        iv. What sorts of issues do you think *should* come to your attention but don’t?
    b. Tell me about the first office visit following hospitalization.
        i. What are your priorities during such a visit? Besides seeing you, does the Veteran see other staff? In particular, is the clinical pharmacist typically involved?
        ii. What is medication reconciliation like at that visit?
        iii. Are there special issues related to medications that are on your mind during that visit?

Perspectives on Medication Reconciliation and Adverse Drug Events

1. What does medication reconciliation, or “med rec” mean for you?
2. Is there a standard protocol in your clinic by which med rec is achieved?
3. What is the utility of med rec, if any and why?

4. What proportion of your patient panel do you believe are confident and accurate in managing their medications?
5. In an ideal world, if you had unlimited resources at your discretion, tell me how you think medication reconciliation should be done performed. What would be the gold standard for med rec?
    a. Who does it? Frequency? How is the discussion initiated? Allocated time? Physical pill viewing?
6. Tell me about your perception of adverse drug events.
    a. In your practice, how frequently do you see medication-related adverse drug events?
    b. Are adverse drug events a significant cause of morbidity/mortality for your patients?
    c. What decision making/support tools do you have that help you prevent adverse drug events?
    d. Are adverse drug events discussed amongst your colleagues?
    e. Does your practice track adverse drug event occurrences? [If yes] How are adverse drug events recorded and reported?

Medication Reconciliation Practice

1. For any given patient, what do you think is the average number of times they will undergo med rec every year? (ball park estimate is fine) Are there set events or visit types that trigger med rec e.g. immediately after discharge, annual visit, problem visit, every visit?
2. Do patients ever come to your clinic for the sole purpose of a med rec visit? If so, how frequently does this happen? What is the selection criterion for these patients?
3. What other staff and clinicians are involved in med rec? Nurses? Pharmacists? Health techs? Others?
4. What percent of patients have their med rec performed before they see you? Tell me about how this is done.
   a. Who completes the med rec? Is there any formal training for med rec technique that these clinicians/staff receive?
   b. Are patients instructed/reminded to bring their medications/list of medications? If so how does this happen?
   c. Is the patient prompted with the name of meds? Or does the patient state their meds? Are the physical pills viewed?
   d. If a discrepancy is noted, what happens? Is there any follow up with the patient after they leave clinic?
5. Tell me about your own approach to med rec.

a. In what instances do you elicit an updated medication list during a patient encounter?
b. What is your approach to med rec for Veterans who are elderly, have multiple chronic illnesses, or cognitive impairment?
c. Are there certain cases/types of patients/situations where you forgo performing med rec If so, why?
d. What are some specific tools that you've used to guide your approach to med rec? This can include any CME training you have received, your own assessment of the literature, expert guidelines, etc.
e. How long does the general med rec take? Do you specifically allocate time for the med rec during a patient visit?
f. Do you have the patient tell you what medications they take? Is the patient prompted with names of their meds and dosing regimen and asked to confirm? Or are the physical pills viewed? How is the family involved in the process?
g. What percent of the time are the physical medications present?
h. For each medication, what information is verified? (Drug, dosing schedule, route)
i. Are supplements included in the medication list?
j. Have you ever received formal training on the medication reconciliation process?

6. If you had unlimited resources, what would you change about the way med rec is performed by you and/or your staff?

7. Why is there a gap between how you envision ideal medication reconciliation and how it is performed? What can be changed at the VA level to get us closer to the ideal med rec?
8. How effective is med rec, in its current form, in preventing ADE? How effective would med rec in its "gold standard" form be in preventing ADE?

Secure Messaging

I want to shift gears now and talk about Secure Messaging. We'll get back to med rec in a moment.

1. Tell me about your understanding of the secure messaging service.
   a. Where do you access it? Who can you reach through the system? What content is meant to be communicated via this system versus general email?
2. How frequently do you use the system? How often do you use it to communicate with patients?
3. What happens when a Veteran sends a message to you?
   a. Who first retrieves the message?
   b. Does every message get to you? If not, which ones are "filtered" out?
   c. How do staff inform you that they are sending a message to you, or leaving one for you to handle?

4. What are the biggest barriers that prevent you from using the system more frequently? What is ineffective or frustrating about the system? What are the most useful dimensions of the system that you can see?
5. How could secure messaging be used for medication reconciliation?
6. Imagine a system where Veterans recently discharged from a hospital stay could self-reconcile medications by viewing pictures of their medications in a secure message via MyHealth*e*Vet; this would then trigger a Pharmacist-reviewed updated medication list in CPRS.
   a. Would med rec through such a system be useful to you in your practice?
   b. What effect do you think it would have on ADEs?
   c. How would you like to be notified about med rec via such a system? Would you like to be notified about all med rec for all Veterans or only the ones where a discrepancy is noted?
   d. In your opinion, who is the appropriate clinician or staff member to whom this information should be sent?

Thank you!

Is there anything else you would like to say about anything we discussed today?

Thank you for your time and participation. Your input is extremely valuable.
