# Supplementary material for: Aligning Medication Reconciliation and Secure Messaging: Qualitative Study of Primary Care Providers’ Perspectives
Source: J Med Internet Res. 2013 Dec 2;15(12):e264. doi: 10.2196/jmir.2793 (PMC3868963; doi:10.2196/jmir.2793)
Supplement: Supplementary file 3 [file jmir_v15i12e264_app3.pdf]

Coding Scheme

Code	Sub Code	Sub Code	# of Interviews	Total # of times Coded
Adverse Drug Events			15	46
	Discussion		15	16
	Occurrence		15	19
	Prevention		15	21
	Reporting		15	16
	Tools and Training		15	17
Clinic			15	56
	Care Team Structure		15	20
	Location		15	15
	Patient Population		15	43
		Age	15	17
		Distribution	15	16
		Gender	15	17
		Hospitalized	15	24
	Workload		15	16
Communication-Hospitalization			15	25
	Patient Admission		15	18

	Patient Discharge	15	19
	Time	9	10
Follow Up Visit		15	24
	Priorities and Other	15	26
	Providers	14	16
Gender Specific Care			
Ideal Med Rec		15	47
	Chasm	14	16
	Suggestions	15	30
Intervention		15	25
	Adverse Drug Events	14	14
	Communication	15	16
	Med Rec Efficiency	9	11
Med Rec Challenges		11	29
	Dual Care	4	5
	Institutional	6	15
	Patient	8	18
Med Rec-Patient		15	94
	Accuracy	15	16
	Approach-Cognitively	15	20

	Impaired		
	General Approach	15	81
Med Rec-General		15	133
	Definition	15	15
	Formal Training and Tools	15	36
	Standard Protocol and Workflow	15	69
	Time Taken	15	25
	Utility	15	17
PACT			
Pharmacy			
Secure Messaging		15	36
	Barriers	14	18
	Benefits	13	13
	Content	11	13
	Frequency	12	13
	Workflow	14	25
Transition		15	28
	Communication	14	16
	Problems- At Appointment	15	15
	Problems- Should but not	13	13
	Scheduling	14	16

Workflow		15	32
	Involvement (inpatient)	14	15
	Visit Structure	14	15
